# Effect of Low Cycle Fatigue Predamage on Very High Cycle Fatigue Behavior of TC21 Titanium Alloy

**DOI:** 10.3390/ma10121384

**Published:** 2017-12-04

**Authors:** Baohua Nie, Zihua Zhao, Yongzhong Ouyang, Dongchu Chen, Hong Chen, Haibo Sun, Shu Liu

**Affiliations:** 1 School of Materials Science and Energy Engineering, Foshan University, Foshan 528000, China; niebaohua121@163.com (B.N.); chendc@fosu.edu.cn (D.C.); chenhongcs@126.com (H.C.); sunmyseven@126.com (H.S.); liushu814194678@126.com (S.L.); 2 School of Materials Science and Engineering, Beihang University, Beijing 100191, China; 3 School of Environmental and Chemical Engineering, Foshan University, Foshan 528000, China; ouyang7492@163.com

**Keywords:** very high cycle fatigue, fatigue predamage, titanium alloy, LCF

## Abstract

The effect of low cycle fatigue (LCF) predamage on the subsequent very high cycle fatigue (VHCF) behavior is investigated in TC21 titanium alloy. LCF predamage is applied under 1.8% strain amplitude up to various fractions of the expected life and subsequent VHCF properties are determined using ultrasonic fatigue tests. Results show that 5% of predamage insignificantly affects the VHCF limit due to the absence of precrack, but decreases the subsequent fatigue crack initiation life estimated by the Pairs law. Precracks introduced by 10% and 20% of predamage significantly reduce the subsequent VHCF limits. The crack initiation site shifts from subsurface-induced fracture for undamaged and 5% predamaged specimens to surface precrack for 10% and 20% predamaged specimens in very high cycle region. Furthermore, the predicted fatigue limits based on the El Haddad modified model for the predamaged specimens agree with the experimental results.

## 1. Introduction

Titanium alloy is widely used for aeronautical structures because of its high specific strength, toughness, and damage tolerance [1]. Throughout the ultra-long time service, the components are subjected to high frequency, low amplitude, and cyclic load; thus, a very high cycle fatigue (VHCF) of titanium alloys in the life regime beyond 10^7^ cycles has been drawing a worldwide attention [2,3]. In practice, aeronautical structures may consist of low cycles fatigue (LCF) resulting from the takeoff and dropdown of the aircraft, and VHCF generated by high-frequency vibrations. A “damage tolerant” design for LCF would be to relate the remaining life based on crack propagation to an inspectable flaw size. However, direct application of such an approach cannot work for “pure” VHCF because the required inspection sizes are well below the state-of-the-art in nondestructive inspection (NDI) techniques. Specially, fatigue damage occurred in specimen subsurface for VHCF is difficult to inspect by the traditional NDI techniques. VHCF requires a relatively large fraction of life for initiation. In addition, crack propagation times to failure could be extremely short due to the high frequencies in VHCF, and the resultant inspection intervals would be too short to be practical. Thus, interest is increasing not only in the capability of pure VHCF but also in that of VHCF combined with LCF fatigue damage.

Recently, few studies have been devoted to the LCF/VHCF combined fatigue behavior of titanium alloys. Hang [4,5] indicated that LCF load significantly decreased the VHCF strength of low carbon-manganese steel, and developed continuum damage mechanics model to evaluate cumulative damage of LCF and VHCF. Mayer [6] showed that the deleterious influence of low load cycles below constant amplitude fatigue limit was underestimated for very high cycle fatigue damage of 100Cr6 steel under variable amplitude (VA) loading condition. However, the effect of LCF load on VHCF fracture mechanism for titanium alloys is not well understood. As for the low carbon-manganese steel, the LCF and VHCF cracks are incline to initiate from the specimens surface, and the fatigue damage accumulation under the LCF/VHCF combined fatigue accelerated the crack initiation. VHCF cracks of high strength titanium alloys mainly induced from the heterogeneous microstructure, such as primary α phase [7,8,9] as well as super grain (grain clusters with similar orientation) [10], whereas LCF cracks are usually initiated from the specimen surface due to the surface machining flaws and persist slip bands. The competition between the LCF damage and materials interior heterogeneous microstructure is focused on LCF/VHCF combined fatigue of high strength titanium alloys.

A practical problem arises in designing against VHCF with the occurrence of high stress transients (LCF loading), which may not lead to failure during the design life, but may degrade the capability of the material regarding its VHCF resistance. Treating VHCF as pure failure modes in fatigue design practice is nonconservative throughout ultra-long life service. In the past years, a Kitagawa–Takahashi (K–T) diagram joined by the El Haddad model can be useful in evaluating the potential for a crack to reduce the HCF capability of a material [11]. The investigations showed that the effect of LCF on HCF limits is affected by not only LCF crack depth, but also the stress ratio R or the residual stress ahead of the crack tip [12]. Cycling at high stress for up to 25% of life has little effect on the HCF strength for Ti6Al4V. However, the HCF strength is reduced by an average of 19% when subjected to prior cycles followed by a stress relief process. Recently, Zerbst [13] modified the El Haddad model based on the cyclic R curve, which can be described by giving the fatigue crack propagation threshold as a function of the crack extension. Compared to HCF, VHCF has lower fatigue limits at 10^9^ cycles, and has lower tolerance to materials defects. It is expected the LCF damage can significantly affect VHCF properties.

The effect of LCF predamage on VHCF behavior of TC21 titanium alloy was investigated in the present work. The LCF up to various fractions of expect life was used to introduce predamage, and then the VHCF behaviors were subsequently studied. This work aimed to enable fundamental understanding of very high cycle fatigue fracture mechanism combined with LCF predamage.

## 2. Experimental Procedures

### 2.1. Materials

**Figure 1:**
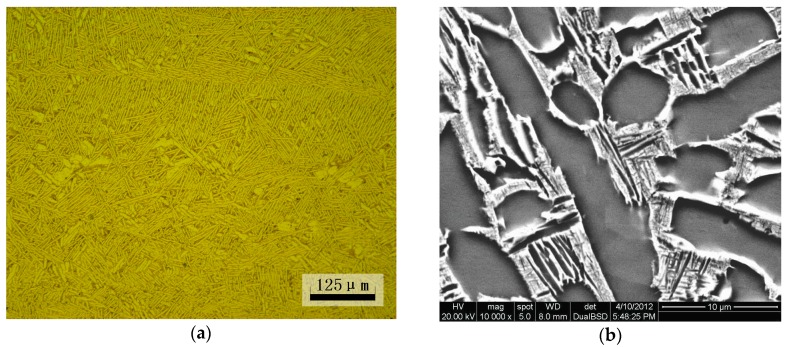
Basketweave microstructures of TC21 titanium alloy: (**a**) optical micrograph and (**b**) backscattered electron micrograph.

The material used in this study was TC21 titanium alloy with a nominal chemical composition of Ti-6Al-2Sn-2Zr-3Mo-1Cr-2Nb. Heat treatment was as follows: 900 °C for 2 h, air quenching, and then 600 °C for 4 h, air quenching. The heat-treated material had a high yield strength of 970 MPa and tensile strength of 1070 MPa. A double lamellar basket weave microstructure was observed (Figure 1).

### 2.2. Surface Treatment

The specimens underwent electropolishing (EP) to remove the machining layers to observe fatigue damage morphology and eliminate its influence on fatigue behavior. Electropolishing was carried out in 59% methanol, 35% *n*-butanol, 6% perchloric acid under −20 °C temperature and 20–25 V voltage.

### 2.3. Fatigue Test

#### 2.3.1. Ultrasonic Fatigue Test

Fatigue tests were carried out using an ultrasonic fatigue test machine (20 kHz) at a constant load ratio of R = −1. The ultrasonic fatigue testing method is an accelerated testing method with a frequency far beyond that of conventional fatigue experiments, which brings advantages of effectiveness and economy morphologies for very high cycle fatigue tests comparing with conventional tests method [14]. An ultrasonic generator transforms 50 or 60 Hz voltage signal into sinusoidal signal with 20 kHz; a piezoelectric converter excited by the generator transforms the electrical signal into longitudinal mechanical vibration with same frequency; an ultrasonic horn amplifies the vibration displacement in order to obtain the required strain amplitude in the middle section of specimen; a computer control system is necessary to control the load amplitude and acquire test data. The maximum displacement amplitude measured by means of a dynamic sensor is obtained at the end of the specimen, while the strain excitation in push–pull cycles (load ratio R = −1) reaches the maximum in the middle section of the specimen, which produces the required high frequency fatigue stress. In addition, a compressed air cooling gun is necessary to be used to prevent the temperature increasing of specimen in the tests.

**Figure 2:**
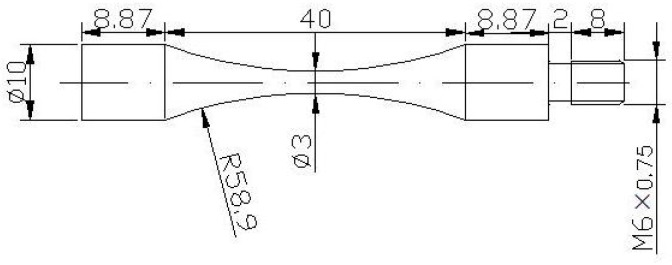
Shape and dimensions of the test specimens.

Considering that the amplifier and the specimen must work at resonance, the specimen geometry was designed using the elastic wave theory. Figure 2 shows the geometries of the fatigue specimens and its dimensions.

#### 2.3.2. LCF Fatigue Tests

The specimen were tested in uni-axial reversed strain amplitude in a conventional hydraulic fatigue machine (Instron 8801, Instron Company, Boston, MA, USA). Considering the dimensions of ultrasonic fatigue test specimens (Figure 2), a LCF test was controlled under transverse diameter deformation. The diameter deformation strain ε*_dia_* can be converted to longitudinal plastic strain ε*_p_* and longitudinal total strain ε through the formulas below [4]:

(1) $$\varepsilon_{p}=-\frac{1}{\nu_{p}}(\varepsilon_{dia}+\nu_{e}\frac{\sigma }{E})$$

(2) $$\varepsilon =\frac{\sigma }{E}(1-\frac{\nu_{e}}{\nu_{p}})-\frac{1}{\nu_{p}}\varepsilon_{dia}$$

where *E*, σ, ν*_e_* and ν*_p_* are the Young modulus, longitudinal stress, elastic Poisson coefficient and plastic Poisson coefficient and its value is always 0.5, respectively.

**Figure 3:**
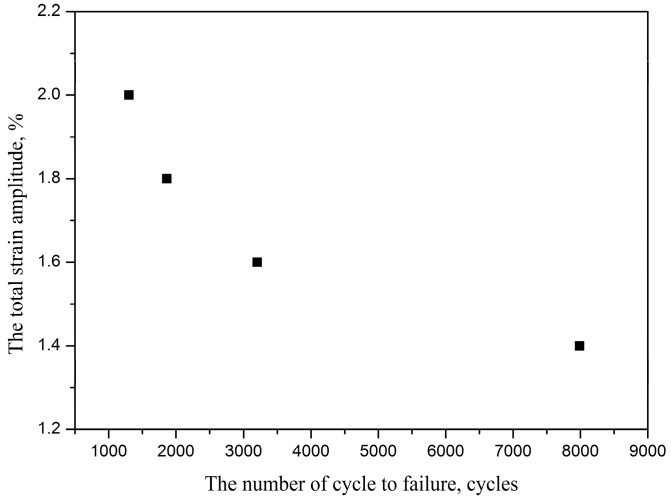
Cycle strain-life of TC21 titanium alloy.

In all LCF tests, a reversed triangle strain waveform was submitted to the specimens. The failure condition is set as its maximum stress decreasing 20% after its cyclic saturation. Figure 3 shows the fatigue life is 1864 cycles at 1.8% strain amplitude. Furthermore, the fatigue damage morphology of different stage fatigue were observed using a video microscope.

#### 2.3.3. LCF/VHCF Combined Fatigue Tests

In order to investigate the effect of LCF predamage on behavior of VHCF for TC21 titanium alloy, specimens were submitted to a same prior 1.8% strain range (strain ratio: −1). LCF predamage was applied onto ultrasonic fatigue specimens at 1.8% strain range for 90 cycles (5% of fatigue life), 180 cycles (10% of fatigue life), 360 cycles (20% of fatigue life), respectively. The subsequent VHCF tests are performed by using ultrasonic fatigue test machine at R = −1, room temperature.

#### 2.3.4. Fatigue Precrack Propagation

Fatigue precrack was obtained by LCF at 1.8% strain range and the ratio of −1. The propagation of precrack under subsequently low stress amplitude were observed using a video microscope. Fatigue crack propagation rate was expressed as follows:

(3) $$\text{d}a/\text{d}N=\frac{\Delta a}{\Delta N}=\frac{a_{i+1}-a_{i}}{N_{i+1}-N_{i}}$$

where $a_{i}$ is crack depth at cycle number *N_i_*, which is supposed to be equal to 0.8c [15], and a surface crack with length 2c was obtained using the video microscope.

## 3. Results

### 3.1. LCF Damage of TC21 Titanium Alloy

**Figure 4:**
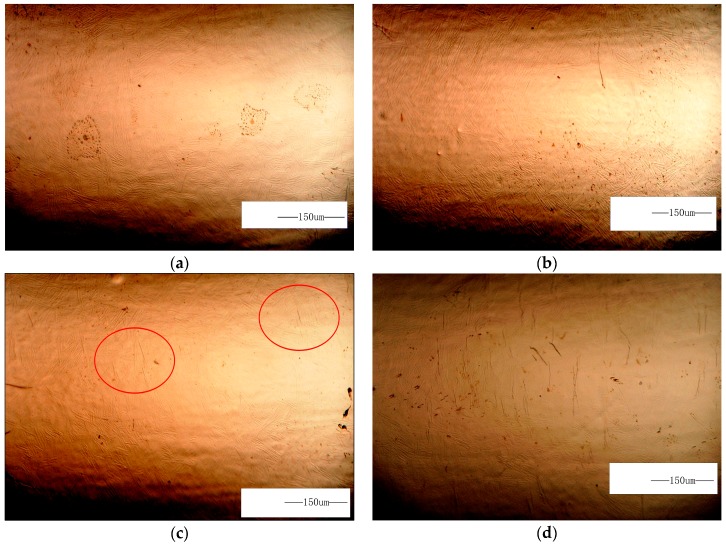
LCF surface damage evolution of TC21 titanium alloy under 1.8% strain range: (**a**) *N* = 0 cycles; (**b**) *N* = 90 cycles (5%); (**c**) *N* = 180 cycles (10%); (**d**) *N* = 360 cycles (20%).

The LCF surface damage evolution of TC21 titanium alloy under 1.8% strain amplitude is shown in Figure 4. No surface crack is observed in 90 cycles (5% of LCF life) until the number of cycles increase to 180 cycles (about 10% of LCF life). However, the number of microcrack significantly increase in 360 cycles (about 20% of LCF life), then these microcrack were expanded to merge until the specimen is broken.

**Figure 5:**
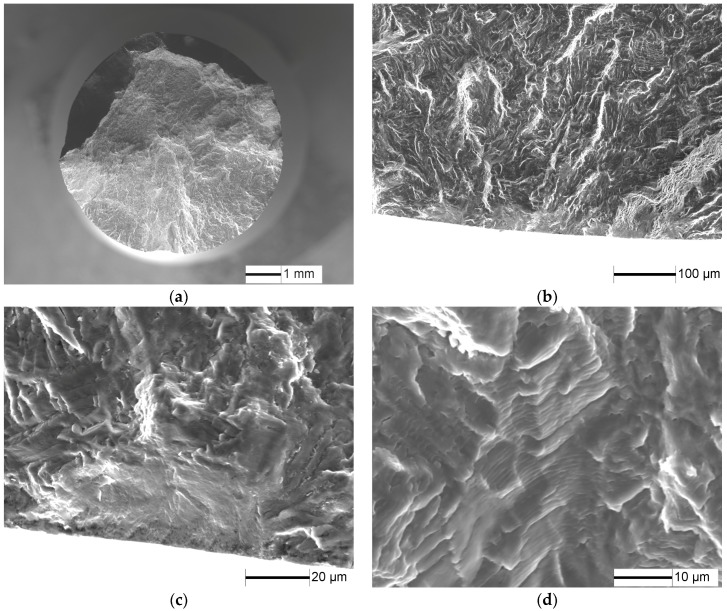
Fatigue fracture surface of TC21 alloy under 1.8% strain range, *N* = 1864 cycles: (**a**) macroscopic morphology; (**b**) crack initiation morphology; (**c**) crack initiation morphology; and (**d**) crack propagation morphology.

The fracture surfaces of specimens under 1.8% strain range, *N*_f_ = 1864 cycles are shown in Figure 5. LCF cracks initiate from multiple sites on the sample surface, and a radial ridge pattern parallel to the crack propagation direction is observed on the fracture surface (Figure 5a). Some small elliptical planes have traces of friction at the fatigue crack initiation site (Figure 5b,c), indicating that these small cracks were expanded to merge to fracture, in accordance with Figure 4d. Cleavage morphology is observed near the small planes (Figure 5c) and typical fatigue striation is displayed on fatigue crack steady propagation (Figure 5d).

### 3.2. S-N Curves After Fatigue Predamage

**Figure 6:**
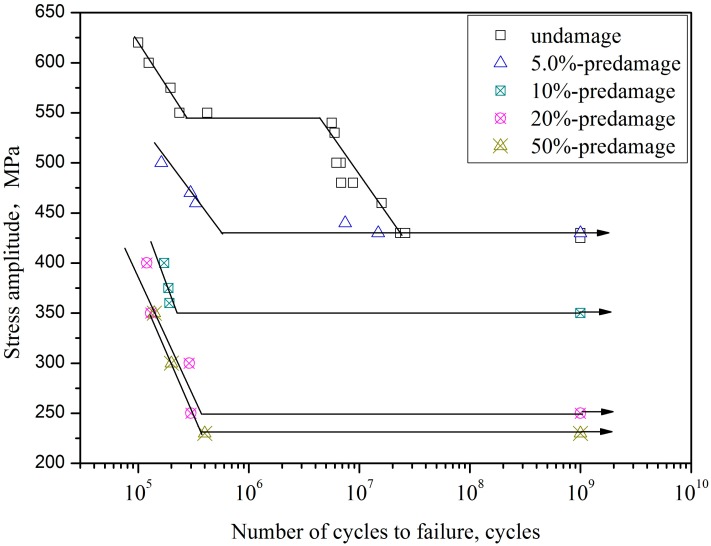
*S*-*N* curves of TC21 for predamaged specimens (Arrows denote the run-out specimens).

Figure 6 shows that the *S*-*N* curve of undamaged specimens exhibit a stepwise shape, which is similar to the references [16,17]. However, in the predamaged specimens, there is a knee of horizontal lines for their *S*-*N* curves in the regime above 10^5^ cycles. Five percent of LCF predamage insignificantly affects the fatigue limit but remarkably decreases fatigue life above fatigue limit compared with undamaged specimens. Fatigue life at 500 MPa stress amplitude is reduced by two orders of magnitude after 5% of LCF predamage. Fatigue limits for 10%, 20% and 50% of the expected life predamage decrease from 430 MPa to 350 MPa, 250 MPa and 230 MPa for the undamaged specimens, respectively, and the fatigue limit of 20% predamage decreases up to 42%, indicating that treating VHCF as pure failure modes in fatigue design practice is nonconservative throughout ultra-long life service. It is should be noted that the decrease effect due to LCF for TC21 titanium alloy is stronger than its for A42 steel [4,5] in VHCF, which can be attributed to the stronger decrease effect in VHCF for high strength titanium alloys [9]. However, it was reported that the LCF predamage with high R value insignificantly affects fatigue limit because of its overloading effect [18,19]. In this paper, residual compressive stress is introduced at the precrack tip because of tensile overload; however, the precrack that acts as a blunt notch yields a residual tensile stress due to the high compression overload for a stress ratio of −1. Thus, the overloading effect of the alloy is significantly reduced by the residual tensile stress.

### 3.3. SEM Observation of the Fracture Surface

**Figure 7:**
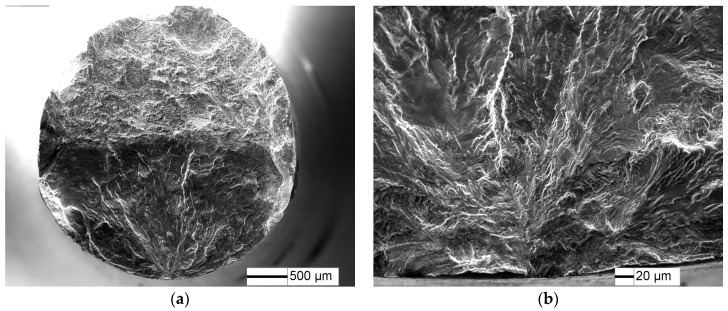
Fatigue fracture surface of TC21 titanium alloy at σ = 550 MPa and *N* = 2.37 × 10^5^ cycles: (**a**) fatigue crack initiation site; and (**b**) high magnification morphology of crack initiation site.

**Figure 8:**
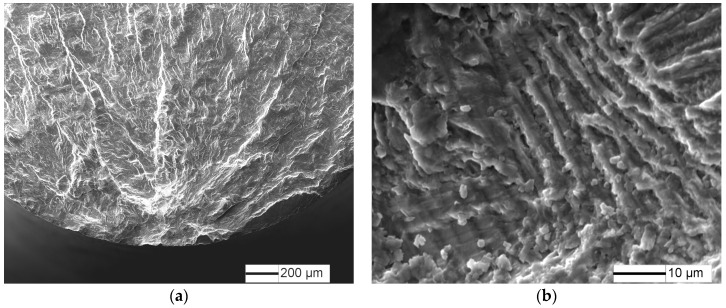
Fatigue fracture surface of TC21 titanium alloy at σ = 480 MPa and *N* = 6.86 × 10^6^ cycles: (**a**) fatigue crack initiation site, and (**b**) high magnification morphology of crack initiation site.

As is showed in Figure 7, fatigue crack of TC21 titanium alloy in the less than 10^6^ cycles region initiates from the sample surface. However, subsurface crack initiation occurs in longer than 10^6^ cycles. α/β lamellar morphology are observed at the crack initiation site where fine granular area (FGA) is found along the α lamellar (Figure 8). The FGA morphology of TC21 titanium alloy are similar to those of high-strength steels [20], although nonmetallic inclusions are not observed at the FGA center.

**Figure 9:**
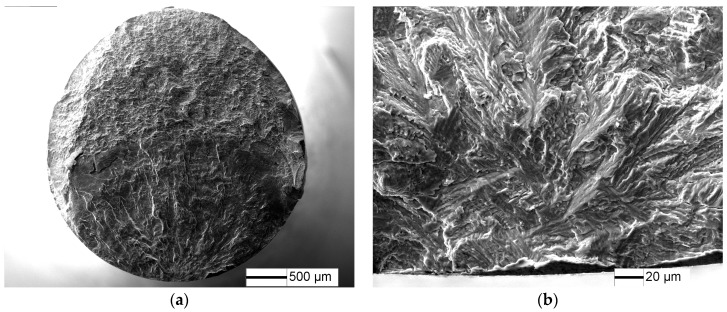
Fracture surface for 5% predamaged specimens at σ = 470 MPa and *N* = 1.27 × 10^5^ cycles: (**a**) macroscopic morphology; and (**b**) crack initiation morphology.

**Figure 10:**
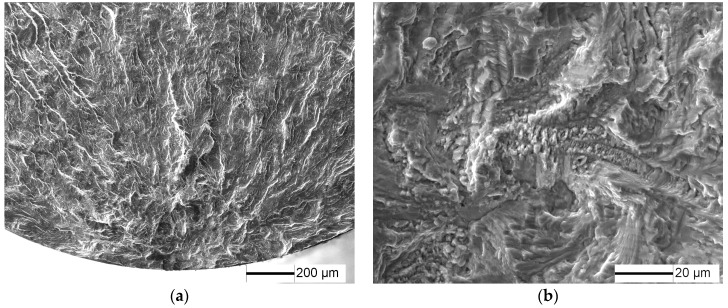
Fracture surface for 5% predamaged specimens at σ = 430 MPa and *N* = 1.48 × 10^7^ cycles: (**a**) macroscopic morphology; and (**b**) crack initiation morphology.

The typical fatigue fracture surfaces of the 5% predamaged specimens are shown in Figure 9 and Figure 10. Fatigue crack initiates from the specimen surface at 470 MPa stress amplitude (Figure 10), while the fatigue crack tends to initiate from the specimen subsurface for an undamaged specimen (Figure 8). Radial ridge pattern parallel to the crack propagation direction is displayed on the fracture surface. However, fatigue crack initiates from the specimen subsurface at VHCF limit stress amplitude (430 MPa), and a fine granular area is observed at the crack initiation site (Figure 10), which has similar crack initiation morphology to undamaged specimens.

**Figure 11:**
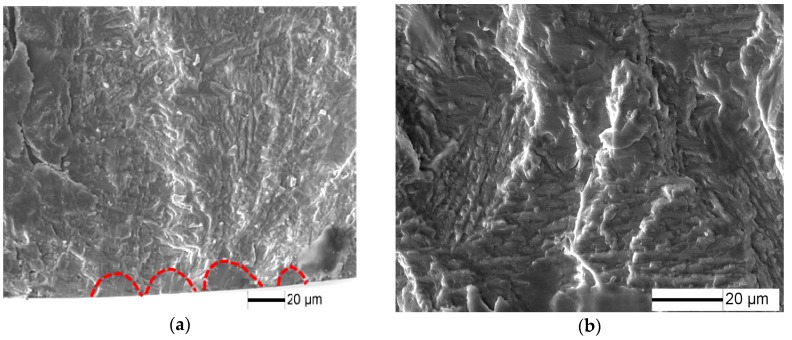
Fracture surface for 10% predamaged specimens at σ = 375 MPa and *N* = 1.88 × 10^5^ cycles: (**a**) crack initiation morphology; and (**b**) crack propagation morphology

**Figure 12:**
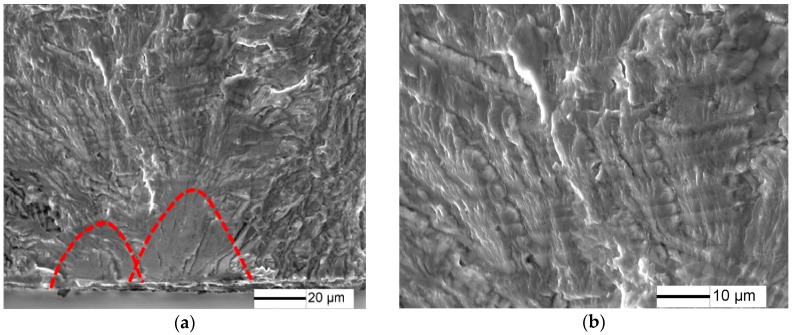
Fracture surface for 20% predamaged specimens at σ = 350 MPa and *N* = 1.35 × 10^5^ cycles: (**a**) crack initiation morphology; and (**b**) crack propagation morphology.

**Figure 13:**
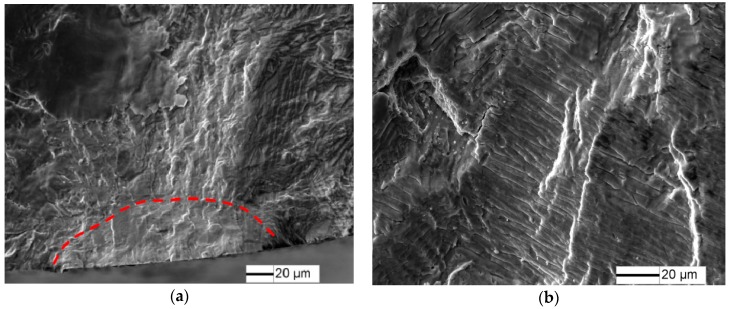
Fracture surface for 50% predamaged specimens at σ = 230 MPa and *N* = 4 × 10^5^ cycles: (**a**) crack initiation morphology; and (**b**) crack propagation morphology.

Fatigue fracture surfaces of 10%, 20% and 50% predamaged specimens at low stress amplitude are shown in Figure 11, Figure 12 and Figure 13. Fatigue crack initiates from the specimen surface, and a small elliptical plane with traces of friction is observed at the fatigue crack initiation site (Figure 11a, Figure 12a and Figure 13a), similar to that of LCF crack. The depth of the small plane is approximately 14.7 µm, 41.5 µm and 49.3 µm, respectively. Moreover, crack-propagation morphology is observed outside the small plane at the crack initiation site. The crack-propagation morphology is associated with α/β lamellar microstructure generated by the subsequent low stress fatigue (Figure 11b, Figure 12b and Figure 13b), rather than the cleavage morphology generated by LCF. Therefore, the small plane at the crack initiation site is supposed to be formed from fatigue precracks.

## 4. Discussion

### 4.1. Effect of LCF Predamage on VHCF Fracture Mechanism

As for TC21 titanium alloy, the fatigue crack initiates from α lamellar (Figure 8) in the sample interior above 10^6^ cycles. However, the VHCF crack initiation mechanism of TC21 titanium alloy with LCF predamage is depended on the LCF predamage and the subsequent stress amplitude in VHCF. For 5% of predamage, the fatigue precrack is not formed by the fatigue predamage, however, it reduces the crack initiation phase and then decreases its fatigue life. As for the 1.8% strain amplitude, the corresponding stress can be approached to yield stress, and plastic deformation accumulation takes place during the LCF predamage. Thus, the slip systems can be activated by plastic deformation accumulation [21,22]. According to the weaken chain theory, a fatigue predamage site is a weak point, and then high cycle fatigue cracks can initiate from the predamage site due to the activating slip systems. However, a fatigue crack initiates from the specimen subsurface at low stress amplitude for 5% predamage specimens, indicating that fatigue early damage does not promote the surface crack initiation at low stress amplitude.

As precracks are introduced by LCF predamage, afatigue crack can be initiated from the precracks under subsequent low stress amplitude. In other words, the precracks can be restarted under low stress amplitude (Figure 11, Figure 12 and Figure 13). The effect of precracks on the fatigue limits can be estimated by El Haddad model. Recently, Zerbst [13] modified the El Haddad model and the Kitagawa–Takahashi diagram. The endurance limits stress range Δσ is obtained as:

(4) $$\Delta \sigma =\frac{\Delta K_{th}(\Delta a)}{Y(a_{i}+\Delta a)\sqrt{\pi (a_{i}+\Delta a)}}$$

which is the mathematical description of the K–T diagram. Δσ is the stress amplitude range. In ultrasonic fatigue with a mean load equal to zero (R = −1), only the tensile part of the cycle has a predominant effect on the fatigue crack propagation [23]. Δσ is replaced by stress amplitude $\sigma_{a}$. The geometry factor *Y* is equal to 0.728 for small semicircular surface cracks [13]. $a_{i}$ and Δa are the initial closure-free crack size and the crack, and fatigue crack $a=a_{i}+\Delta a$.

El Haddad’s $\Delta K_{th}(\Delta a)$ equation has to be modified by adding an additional term $a^{*}$:

(5) $$\Delta K_{th}(\Delta a)=\Delta K_{th,LC}\sqrt{\frac{\Delta a+a^{*}}{\Delta a+a^{*}+a_{0}}}$$

The additional $a^{*}$ is simply determined by:

(6) $$a^{*}=a_{0}\frac{{\left(\Delta K_{th,eff}/\Delta K_{th,LC}\right)}^{2}}{1-{\left(\Delta K_{th,eff}/\Delta K_{th,LC}\right)}^{2}}$$

The intrinsic fatigue propagation threshold, $\Delta K_{th,eff}$, can be estimated by $\Delta K_{th,eff}=E\sqrt{b}$ [24]. The term $a^{*}$ is introduced to fulfill the condition that $\Delta K_{th}=\Delta K_{th,eff}$ for Δa = 0. The intrinsic crack length $a_{0}$ is given by [11]:

(7) $$a_{0}=\frac{1}{\pi }{\left(\frac{\Delta K_{th,LC}}{Y\Delta \sigma_{e}}\right)}^{2}$$

In the absence of large defects, the initial closure-free crack *a_i_* can be referred as the arrested microstructurally short crack *d*_1_, which is given as:

(8) $$d_{1}=\frac{1}{\pi }{\left(\frac{\Delta K_{th,eff}}{Y\Delta \sigma_{e}}\right)}^{2}$$

The intrinsic fatigue propagation threshold, $\Delta K_{th,eff}$ of TC21 alloy is calculated as 2.2 $\mathrm{MPa}\sqrt{m}$. The long crack propagation threshold of TC21 alloy is estimated about 2.8 $\mathrm{MPa}\sqrt{m}$ [25], which is approach to the crack propagation threshold of Ti6Al4V [23]. It should be noted that $\Delta K_{th}$ is not much higher than $\Delta K_{th,eff}$, which can be explained by the very small closure effect when only the tensile part of the cycle load is considered at R = −1.

**Figure 14:**
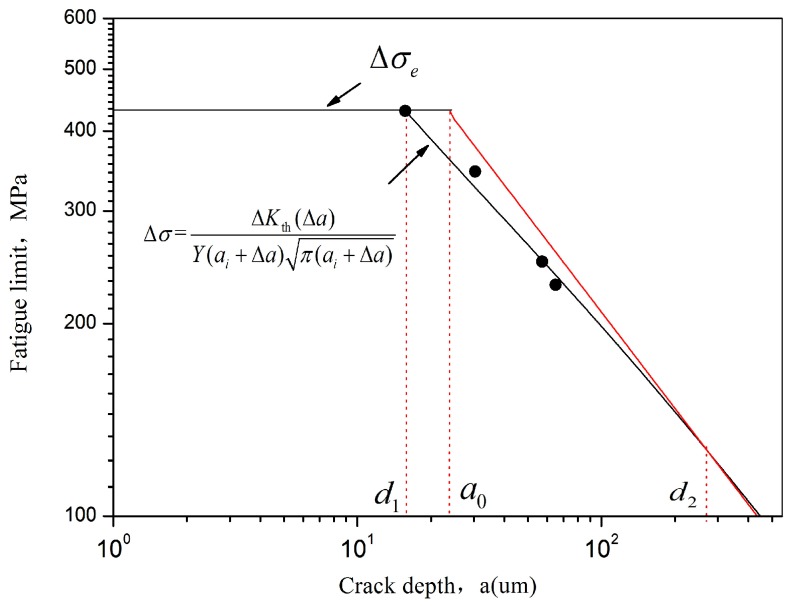
Kitagawa–Takahashi diagram of TC21 titanium alloy with predamage.

The term of *d_i_*, $a_{0}$, $a^{*}$ are calculated as 15.4 µm, 25.4 µm and 40.98 µm, respectively. The K–T diagram is shown in Figure 14. The precrack with the approximately 14.7 µm, 41.5 µm and 49.3 µm depth are introduced by 10%, 20% and 50% fatigue damage. According to Equation (4), the fatigue limit of TC21 alloy with 50% and 20% of predamage is about 230 MPa and 250 MPa, which well agrees with the experimental data (Figure 14). For 10% of predamage, the fatigue limit is estimated as 326 MPa with 6.8% error compared with the experimental data. This finding suggests that fatigue precrack plays a significant role in the reduction of fatigue strength. It is also indicated that the crack depth for the transition between short and long cracks is about 270 µm. 

Furthermore, the cyclic plastic zone at the applied load level, and ω* can be estimated by Tirosh and Peles [26]:

(9) $$\omega^{*}=\frac{1}{30\pi }{\left(\frac{K_{I}}{\sigma_{y}}\right)}^{2}$$

For 10% of predamage, the stress intensity factor under stress amplitude 375 MPa is about 3.02 $\mathrm{MPa}\sqrt{m}$, and the plastic zone is 100 nm, which is smaller than the size of lamellar basketweave. This suggest that fatigue early crack growth is significantly influenced by the microstructure (Figure 12b), and fatigue crack is prone to grow towards the most preferred direction.

### 4.2. Effect of Fatigue Predamage on Fatigue Life

**Figure 15:**
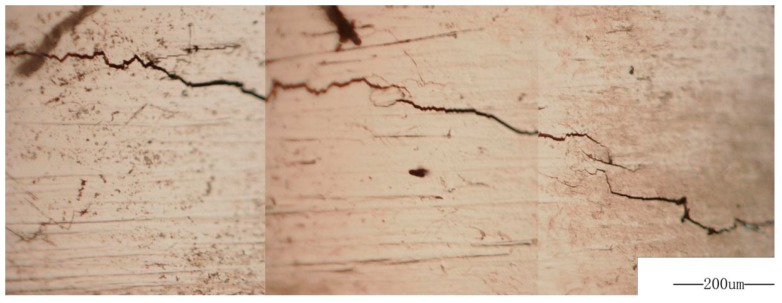
Morphology of fatigue precracks propagation.

**Figure 16:**
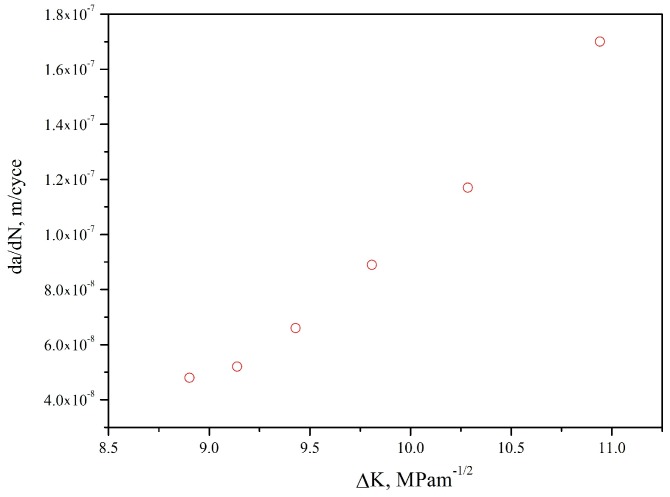
Relation between the expected precrack propagation rate d*a*/d*N* and stress intensity factor ∆*K*.

Fatigue precrack was obtained by LCF at 1.8% strain range and the ratio of −1. The propagation of precrack under a subsequently low stress amplitude were observed using a video microscope showed in Figure 15. The precrack propagation under a low stress amplitude follows Paris law (Figure 16) can be expressed as:

(10) $$da/dN=8.64\times 10^{-13}{\left(\Delta K\right)}^{5.066}$$

The precrack propagation life under a low stress amplitude can be estimated as:

(11) $$N_{p}=\frac{\left(a_{fr}^{1-m/2}-a_{0}^{1-m/2}\right)}{[1-(m/2)]C{\left(Y\Delta \sigma \sqrt{\pi }\right)}^{m}}$$

where $a_{fr}$ is the size of the fatigue fracture zone. C and m can be obtained by Equation (10).

The fatigue precrack acted as a small crack has a higher propagation rate than that of a long crack with the same nominal stress intensity factor range. An intrinsic crack length $a_{0}$ is added to the actual length of crack to unify the differences in the crack propagation rates between small and long cracks [26]. The crack propagation rate independent of crack size can be calculated by linear elastic fracture mechanics.

According to Equation (11), the crack propagation life of TC21 alloy for 5%, 10% and 20% LCF of predamage are shown in Table 1. The precrack propagation lives for 10% and 20% of LCF predamage samples account for a major portion of the expected life, which also indicate that the precrack directly propagate under subsequent stress amplitude. The number of cycles to failure for 10% predamage specimens under 350 MPa is about 2 × 10^5^ cycles, which shows that crack propagation times to failure could be extremely short due to the high frequencies in VHCF. Furthermore, the effect of high frequencies and low stress cannot be ignored for the structure with LCF predamage, although the stress amplitude is much lower than VHCF limits, which is consistent with Mayer’s research [6].

For 5% of LCF predamage specimens, where precracks are not introduced by LCF predamage, more than 20% of the expected life is consumed in the crack-propagation life. Considering that less than 1% of the expected life is consumed in the crack-propagation life for TC21 alloy without predamage. It is indicated that 5% of LCF predamage promote the initiation of fatigue crack.

## 5. Conclusions

The effect of low cycle fatigue (LCF) predamage on the subsequent very high cycle fatigue (VHCF) behavior is investigated in TC21 titanium alloy. The S-N curves of predamaged specimens exsit a knee of horizontal lines for 10^5^~10^9^ cycles. The crack initiation site shifts from subsurface-induced fracture for undamaged and 5% predamaged specimens to surface precrack for 10% and 20% predamaged specimens. Five percent predamage insignificantly affects the VHCF limit due to the absence of precrack, but decreases the subsequent fatigue crack initiation life estimated by the Pairs law. Ten percent and 20% predamage samples account for a major portion of the expected propagation life. Furthermore, the precracks introduced by 10% and 20% predamage significantly reduce the subsequent VHCF limits, which are well-predicted based on the El Haddad modified model.

## Figures and Tables

**Table 1:** Estimation of fatigue crack-propagation life.

Predamaged Specimens	Stress Amplitude, MPa	*N*, Cycles	$a_{0}$, µm	$a_{fr}$, µm	*N*p, Cycles	*N*p/*N*, %
0%	460	1.590 × 10^7^	27.56	1487	8.008 × 10^4^	0.50
5%	460	3.320 × 10^5^	27.56	1487	8.008 × 10^4^	24.12
10%	350	1.920 × 10^5^	42.26	2427	1.440 × 10^5^	74.99
20%	300	2.000 × 10^5^	69.06	2650	1.706 × 10^5^	85.30
